# *Ex vivo* gene modification therapy for genetic skin diseases—recent advances in gene modification technologies and delivery

**DOI:** 10.1111/exd.14314

**Published:** 2021-03-11

**Authors:** Vignesh Jayarajan, Evangelia Kounatidou, Waseem Qasim, Wei‐Li Di

**Affiliations:** ^1^ Infection, Immunity and Inflammation Research & Teaching Department, Immunobiology Section UCL Great Ormond Street Institute of Child Health London UK; ^2^ Infection, Immunity and Inflammation Research & Teaching Department, Molecular and Cellular Immunology Section UCL Great Ormond Street Institute of Child Health London UK

**Keywords:** epidermal sheet graft, gene editing, genodermatoses, keratinocyte stem cell

## Abstract

Genetic skin diseases, also known as genodermatoses, are inherited disorders affecting skin and constitute a large and heterogeneous group of diseases. While genodermatoses are rare with the prevalence rate of less than 1 in 50,000 – 200,000, they frequently occur at birth or early in life and are generally chronic, severe, and could be life‐threatening. The quality of life of patients and their families are severely compromised by the negative psychosocial impact of disease, physical manifestations, and the lack or loss of autonomy. Currently, there are no curative treatments for these conditions. *Ex vivo* gene modification therapy that involves modification or correction of mutant genes in patients’ cells *in vitro* and then transplanted back to patients to restore functional gene expression has being developed for genodermatoses. In this review, the *ex vivo* gene modification therapy strategies for genodermatoses are reviewed, focusing on current advances in gene modification and correction in patients’ cells and delivery of genetically modified cells to patients with discussions on gene therapy trials which have been performed in this area.

## 1 INTRODUCTION

Genetic skin diseases, also known as genodermatoses, are inherited conditions affecting skin. They constitute a large and heterogeneous group of diseases. Studies have indicated that mutations in over 500 unique genes can cause disorders with a distinct skin phenotype.^1^ Currently, the treatments for genodermatoses are generally limited to management of symptoms.^2^, ^3^ With advances in molecular technologies, a large number of inherited skin diseases have been characterized and this paves the way for the development of gene‐based therapies for serious, devastating and sometimes life‐threatening genodermatoses. By resolving the root cause of genetic skin diseases, gene therapy offers the prospect of long‐lived therapeutic interventions.

Allogenic cell therapy has been performed for patients with severe genetic skin conditions. In this therapy, bone marrow cells (BMCs) or mesenchymal stromal cells (MSCs) or somatic epidermal fibroblasts from healthy donors were transplanted onto patients intravenously or subcutaneously.^4^, ^5^, ^6^, ^7^, ^8^ These therapies provided clinical benefits, but human leukocyte antigen (HLA) matching between donors and patients has always been a challenge. Immunosuppressive preconditioning has to be used in allogenic bone marrow cell therapy to prevent graft‐versus‐host disease (GVHD), a lethal complication of allogeneic bone marrow transplantation where genetically disparate host cells get attacked by the immunocompetent donor T cells.^9^ In addition, studies on the disease of dystrophic epidermolysis bullosa showed that only dysfunctional Type VII Collagen (C7) protein secreted by patients’ epithelial cells could be detected following allogenic BMC or MSC transplantation, suggesting that a short‐term clinical improvement following BMCs or MSCs transplantation was more likely due to an anti‐inflammatory property of these cells.^4^, ^6^, ^7^ In comparison with allogenic gene therapy, *ex vivo* autologous gene modification therapy, where patients’ cells are genetically modified *in vitro* and then grafted or injected back to patients, overcomes the disadvantages seen in allogenic cell therapy and has become a favourite gene therapy strategy for genodermatoses, particularly in those diseases with severe epidermal loss, such as junctional epidermolysis bullosa (JEB) and recessive dystrophic epidermolysis bullosa (RDEB).

Topical delivery or intravenous/intradermal injection of therapeutic vectors directly into the skin or patients’ body, known as *in vivo* gene therapy, has become another attractive therapeutic strategy for genodermatoses. This strategy is more suitable for those skin diseases with skin lesions covering whole body, in which local skin grafts and injections have their limitations. Several topical gene therapies using modified herpes simplex virus (HSV) carrying wild‐type genes such as *COL7A1* for RDEB or transglutaminase 1 (*TGM1*) for autosomal recessive congenital ichthyosis have been developed.^10^, ^11^ The outcomes from *in vivo* phase 1 topical delivery gene therapy were promising and encouraging, although the expressions of therapeutic genes in the host skin/cells were transient due to non‐integrating feature of herpes simplex virus, and multiple administrations of therapeutic vector in this *in vivo* gene therapy are required. In addition, topically delivered vectors are difficult to target keratinocyte stem cells (KSCs) as these cells are scattered in the lowest layer of the epidermis. Without targeting long‐lived stem cells, the durability of therapeutic effect is limited. There were few reported *in vivo* gene therapy studies using intravenous delivery approach for genodermatoses as extensive studies are required to demonstrate the risk and safety issues related to vectors through circulation system and the efficiency of therapeutic vectors homing to the skin.

Skin is an attractive target for gene therapy. The epidermis can be readily harvested and manipulated *ex vivo*, and the effects of the therapy can be easily monitored. Advances in culture techniques have allowed keratinocytes including KSCs or holoclones to be cultured *in vitro*.^12^, ^13^, ^14^ Sheets of epithelium can be generated and have primarily been used as autografts in patients with severe burns for more than three decades.^15^ All this has led to the development of *ex vivo* gene‐modified epidermal sheet therapy for genodermatoses. The first *ex vivo* genetically modified epidermal sheet therapy for JEB was reported in 2006 following retroviral transduction of KSCs.^16^ This landmark study provided important proof‐of‐principle evidence that correction of skin stem cells can afford effective therapy for genodermatoses. Since then, a number of *ex vivo* gene‐modified epidermal sheet therapies have been performed. The most remarkable one was the life‐saving epidermal sheet therapy for a JEB patient with complete epidermal loss across 80% of body surface area.^14^ Recently, phase I gene‐modified epidermal sheet therapies for RDEB and Netherton syndrome (NS) were also reported and showed restoration of transgene expressions and functions to some extent^17^, ^18^ (Table 1).

**TABLE 1:** *Ex vivo* epidermal sheet graft therapies discussed in this review.

Disease	Gene	Vector / promoter	Keratinocyte culture / medium	Reference
Junctional epidermolysis Bullosa	*LAMβ3*	Retroviral vector expressing *LAMβ3* cDNA / Moloney Murine Leukaemia Virus Long Terminal Repeat	Primary patient's keratinocytes co‐culture with irradiated 3 T3‐J2 cells/ Rheinwald & Green medium	Mavilio *et al*., 2006^16^
Recessive dystrophic epidermolysis Bullosa	*COL7A1*	Retroviral vector expressing *COL7A1* cDNA / Moloney Murine Leukaemia Virus Long Terminal Repeat	Primary patient's keratinocyte /Serum‐free medium	Siprashvili *et al*., 2016^18^
Junctional epidermolysis Bullosa	*LAMβ3*	Retroviral vector expressing *LAMβ3* *cDNA* / Moloney Murine Leukaemia Virus Long Terminal Repeat	Primary patient's keratinocytes co‐culture with irradiated 3 T3‐J2 cells/ Rheinwald & Green medium	Hirsch *et al*., 2017^14^
Netherton syndrome	*SPINK5*	3^rd^ generation lentiviral expressing codon optimized *SPINK5 cDNA* / Human involucrin promoter	Primary patient's keratinocytes co‐culture with frozen‐irradiated 3 T3‐J2 cells/ Rheinwald & Green medium	Di *et al*., 2019^17^

The success of *ex vivo* genetically modified epidermal sheet therapy relies on the effective gene modification in patients’ KSCs, allowing durable transgene expression in grafted epidermal sheet generated using heterogeneous keratinocyte populations including KSCs. There are, therefore, two challenges in the development of an *ex vivo* genetically modified epidermal sheet therapy, that is correction of mutant genes in primary keratinocytes obtained from patients and retaining KSC stemness in epidermal sheet following gene modification and sheet culture. Several gene modification strategies have been developed. This includes (i) adding a wild‐type gene to patients’ cells harbouring recessive mutations^14^, ^16^, ^17^, ^18^; (ii) introducing antisense oligonucleotides or small interfering RNAs into patients cells/tissues with dominant mutation to silence mutant alleles^19^, ^20^, ^21^; and (iii) editing genomic DNA in patients’ cells using recently developed genome‐editing toolkits such as transcription activator‐like effector nucleases (TALENs), Clustered Regularly Interspaced Short Palindromic Repeats (CRISPR) and CRISPR associated protein 9 (CRISPR/Cas9), programmed base editing, and prime editing.^22^, ^23^, ^24^, ^25^, ^26^, ^27^, ^28^, ^29^ Following gene modification, administration or delivery of genetically modified cells to patients is the next important step for a successful *ex vivo* gene therapy. The common approach to this is to graft epidermal sheets generated using genetically modified patients’ keratinocytes. This requires primary keratinocyte culture *in vitro* to expand cells including KSCs and then grow these cells as epidermal sheets. Several cultivation systems have been used for culturing primary keratinocytes, but the traditional culture system developed by Rheinwald and Green is the only one that has confirmed to retain KSCs population in order to achieve a long‐term survival of the engraftment with durable transgene expression.^14^, ^30^

This review discussed issues in the development of *ex vivo* autologous gene modification therapy for genodermatoses, focusing on gene modification and correction of patients’ cells, administration of gene‐modified cells to patients with discussions on gene therapy trials which have been performed in this area.

### 1.1 Gene‐addition therapy and genome editing using CRISPR/Cas9

Adding an exogenous wild‐type gene into patients’ cells to replace mutant/defective endogenous gene and restore functional gene expression is a common gene modification strategy for recessive genetic skin diseases. The vectors that deliver wild‐type genes into patients’ keratinocytes and fibroblasts are commonly retro‐ and lentiviral vectors.^31^ Both vectors have a transgene packaging capacity of ~8 kb and are able to stably integrate transgenes into host genome.^16^ Retro‐ and lentiviral vectors can also transduce hard‐to‐transfect cells such as keratinocytes with high transduction efficiency and have been used in phase I/II gene therapy trials.^31^ In spite of its success, gene‐addition therapy has its limitations, not least the unregulated expression of genes and ectopic sites of gene insertion. In addition, in some cases, efficiency of transgene transfer has been challenging if the disease‐causing gene is large. For example, mutations in the gene *COL7A1* that causes RDEB are more than 9 kb in size which is more than the packaging capacity of both retro‐ and lentiviral vectors, resulting in low viral titre and transduction efficiency.^32^

Genome editing, aiming to directly repair mutations at genomic level, is therefore more powerful. In early years, genome‐editing toolkits such as Zing‐finger nucleases and TALENs were used to correct mutations in *COL7A1* in keratinocytes.^25^, ^26^, ^33^, ^34^ However, these technologies are not easily adoptable due to either complicated design implicating protein engineering for each target gene or low editing efficiency in certain cells or tissue types.^35^ The recent development of CRISPR/Cas9 technology has revolutionized gene modification approach, bringing a new era in genome editing as it comprises an easy, cheap and universal editing machinery to achieve gene correction. CRISPR relies on the activity of a DNA nuclease Cas9 which can be recruited to the target DNA sequence by a short RNA fragment known as guide RNA (gRNA). A Protospacer Adjacent Motif (PAM) which has to be adjacent to the target DNA sequence is essential for Cas9 binding to exert its DNA cleavage function.^36^ Following DNA cleavage, cellular DNA repair mechanisms are triggered to fix Cas9 induced DNA double‐strand break (DSB). The most prevalent repair mechanism is non‐homologous end joining (NHEJ) in which the broken DNA ends are ligated back together with no regard to homology. NHEJ causes insertions or deletions (indels) of nucleotides that can cause frame shifts/premature termination codons on target gene sites, resulting in gene disruption and knockout, and this genome‐editing strategy has widely been used for generation of disease models with gene knockout.

On the contrary, when a DNA template that shares high degree of homology with the target sequence is provided, homology‐directed repair (HDR) pathway is involved, resulting in precise genetic modifications. This genome‐editing approach that uses CRISPR/Cas9 mediated HDR has been experimentally used for correction of mutant genes in patients’ keratinocytes and fibroblasts with genodermatoses. For example, using the dual sgRNA CRISPR/Cas9 deletion strategy of *COL7A1* exon 80, RDEB keratinocytes harbouring a homozygous carrier of the c.6527insC mutation restored the C7 protein expression.^23^ Studies have also shown correction of a splice‐site mutation (c.425A > G) in exon 3 or a null mutation in exon 2 (c.189delG) of *COL7A1* using CRISPR/Cas9 mediated HDR approach with restoration of the C7 protein expression and anchoring fibril formation.^24^, ^25^ However, it has been noticed that the efficiency of CRISPR/Cas9 mediated HDR was low around 11 ‒ 15%, and this limited CRISPR/Cas9 mediated HDR genome editing for clinical application.^24^ Apart from low editing efficiency, HDR events are often outnumbered by NHEJ, causing a remarkably challenging environment for donor template knock‐in and precise genome editing. Furthermore, the promiscuous activity of Cas9 can cause random DSBs at nonspecific sites (off‐targets), unexpected NHEJ at off‐target sites and induce p53 and apoptosis pathways.^37^, ^38^ All these require further refinements of the conventional CRISPR/Cas9 technology.

### 1.2 Programmed base editing

The approach of programmed single base editing (BE) has been developed recently.^39^ It allows precise, irreversible deamination of one nucleotide to another in a programmable manner, obviating the need for DNA double‐strand break and HDR used in conventional CRISPR/Cas9 genome editing. In the BE toolbox with versions 3 and 4, the conventional Cas9 endonuclease was mutated at point D10A and H840A which are two important residues for endonuclease activity. The changes ultimately resulted in a catalytic deactivation of Cas9, known as dead Cas9 (dCas9) or Cas9 nickase. dCas9 endonuclease tethered to a cytidine deaminase enzyme and uracil DNA‐glycosylase (UGI) element allowed conversion of C•G to T•A by deamination.^40^ Adenine base editors (ABEs) which can convert A•T to G•C have also been developed. In this editing platform, the enzyme apolipoprotein B mRNA editing enzyme catalytic subunit 1 (APOBEC1) in the BE toolbox was replaced with a transfer RNA adenosine deaminase enzyme (TadA).^41^ The combination of BE3/4 and ABE gene‐editing systems can, therefore, potentially mediate all four nucleotide transitions (C > T, A > G, T > C and G > A). Studies have shown that the programmed base editing offered higher efficiencies in genome editing than the conventional CRISPR/Cas9‐HDR (15‒75% vs 0.1‒5%) with much lower off‐target frequencies (<5% for BE and ≤1% for ABE).^42^, ^43^ The development of new versions of ABE including ABEmax, miniABEmax and ABE8e along with ABE8 library has further improved genome‐editing efficiency.^39^, ^44^, ^45^, ^46^ In addition, uridine depleted ABE mRNA combined with the modification of remaining uridine to 5‐methoxyuridine, stabilizes ABE mRNA transcript, increasing dCas9 expression, and reducing immune responses.^47^ All these allow more precise base editing to occur. In CRISPR/Cas9 genome‐editing platform, the canonical PAM sequence NGG adjacent to the target DNA sequence is essential for Cas9 binding. However, the canonical PAM sequence is not always available at the site adjacent to target DNA sequence, and this limits the use of base editing toolboxes. Recently, Hu *et al*. reconfigured Cas9 to xCas9 to expand PAM compatibilities, and studies revealed that xCas9 could recognize a set of varied PAM sequences.^48^ These new versions and modifications of ABE and Cas9 make a better base editing platform for genetic diseases with various gene mutations and locations.

Osborn *et al*. carried out an ABE base editing study in a patient's fibroblasts with skin disease RDEB. These fibrobalasts harboured mutations c.553C>T (R185X) and c.1573 C > T (R525X) in the gene *COL7A1*. Restoration of the endogenous C7 expression was detected following ABE base editing. The correction efficiencies were 24% and 45% for the mutation c.553C>T and 8% and 17% for the mutation c.1573 C > T, confirmed by DNA sequencing and mRNA expression, respectively.^29^ These efficiencies were much higher than conventional CRISPR/Cas9. Nevertheless, base editing also has its limitations. For example, it is unable to correct indel mutations; it requires a PAM sequence adjacent to the target DNA which is not always available; and it promiscuously edits bystander bases when more than one C or A nucleotides are within the editing window, causing potential missense mutations and gene disruption.^49^

### 1.3 The technology of prime editing

Prime editing bypasses the caveats of both conventional CRISPR/Cas9 and programmed base editing by using dCas9 endonuclease fused to an engineered reverse transcriptase, programmed with a prime editing guide RNA (pegRNA) that specifies the target site and encodes the desired edit.^49^ It was reported that the platform could insert up to 44 base pairs (bp) or delete up to 80 bp of nucleotides and correct point mutations including transversions. It can also perform combination edits without making explicit DNA DSBs. Based on this, it has been predicted that prime editing could correct up to ~89% of human genetic variants. The correction of insertion or transversion edits by prime editing has been tested in three human cell lines including K562, U2OS and Hela, and the editing efficiencies in these cell types were between 12% ‐ 30% with low indels of 0.13 – 2.2%. Although the correction seemed cell type dependent, in general, the correction efficiencies were similar or higher compared to CRISPR/Cas9 mediated HDR genome editing with much lower off‐target frequencies.^49^ As prime editing is a newly developed technology, further tests in various cell types for editing efficiency and off‐target effect are necessary, particularly in hard‐to‐transduce cells like keratinocytes. Currently, there are no reports showing the application of prime editing in keratinocytes.

### 1.4 Delivery of genome‐editing toolkit into keratinocytes and fibroblasts

The common approaches to deliver genome‐editing toolkits to patients’ keratinocytes or fibroblasts are viral vectors and electroporation. There was a study showing correction of mutations in *LAMB3* by delivery of Cas9/sgRNA and homologous repair template into keratinocytes using adenoviral vector and integration defective lentiviral vector. However, the efficiency of gene correction was less than 0.5%.^50^ Correction of mutations in *COL7A1* by delivery of sgRNA/Cas9 and homologous repair template to keratinocytes and fibroblasts using defective lentiviral vectors was also reported with the gene‐editing efficiencies of 11% for keratinocytes and 15.7% for fibroblasts.^24^ The reason for a low delivery efficiency by viral vector could be because the sizes of gene‐editing components gRNA, Cas9 cDNA and repair template were too large to be encompassed in a single viral vector, and multiple viral vectors had to be used for gene editing, causing reduced delivery efficiency.^51^

Electroporation with nucleofection reagent has been widely used for delivery of genome‐editing toolkits into cells to overcome the usage of multiple vectors and reduce the risk of genotoxicity. A study on RDEB patients’ cells showed a successful delivery of double‐stranded DNA donor template alongside sgRNA and Cas9 protein (RNP complex) into fibroblasts by electroporation with the delivery efficiency of 33%.^52^ Other studies on RDEB patients or donor cells also reported high gene‐editing efficiency (70‒85%) of delivery RNP gene‐editing complex in keratinocytes using electroporation. They further revealed that electroporated keratinocytes could be used to generate epidermal sheet and graft on human:murine skin graft model to form humanized skin architecture, suggesting retention of stemness in primary keratinocytes after electroporation.^22^, ^53^ This was also supported by another study in which the expression of integrin β1, one of KSC markers, was not changed in primary keratinocytes after electroporation.^54^ All these indicate that electroporation could be a reliable and feasible approach to deliver genome‐editing toolkits into patients’ keratinocytes with higher delivery efficiency and retention of KSC’s stemness. Nevertheless, low cell viability was reported with nearly 20% cell death after electroporation.^54^ To prevent cell death, rho‐associated protein kinase inhibitor (ROCKi) has been widely used in cell culture medium following electroporation. This application is based on the discovery that ROCKi could help in the growth of stem cells and prevent cell death.^55^, ^56^ However, the precise mechanism of ROCKi on cell survival and its cytotoxicity on cells is not clear and needs to be further demonstrated.

### 1.5 Generation of genetically modified epidermal sheet

**FIGURE 1:**
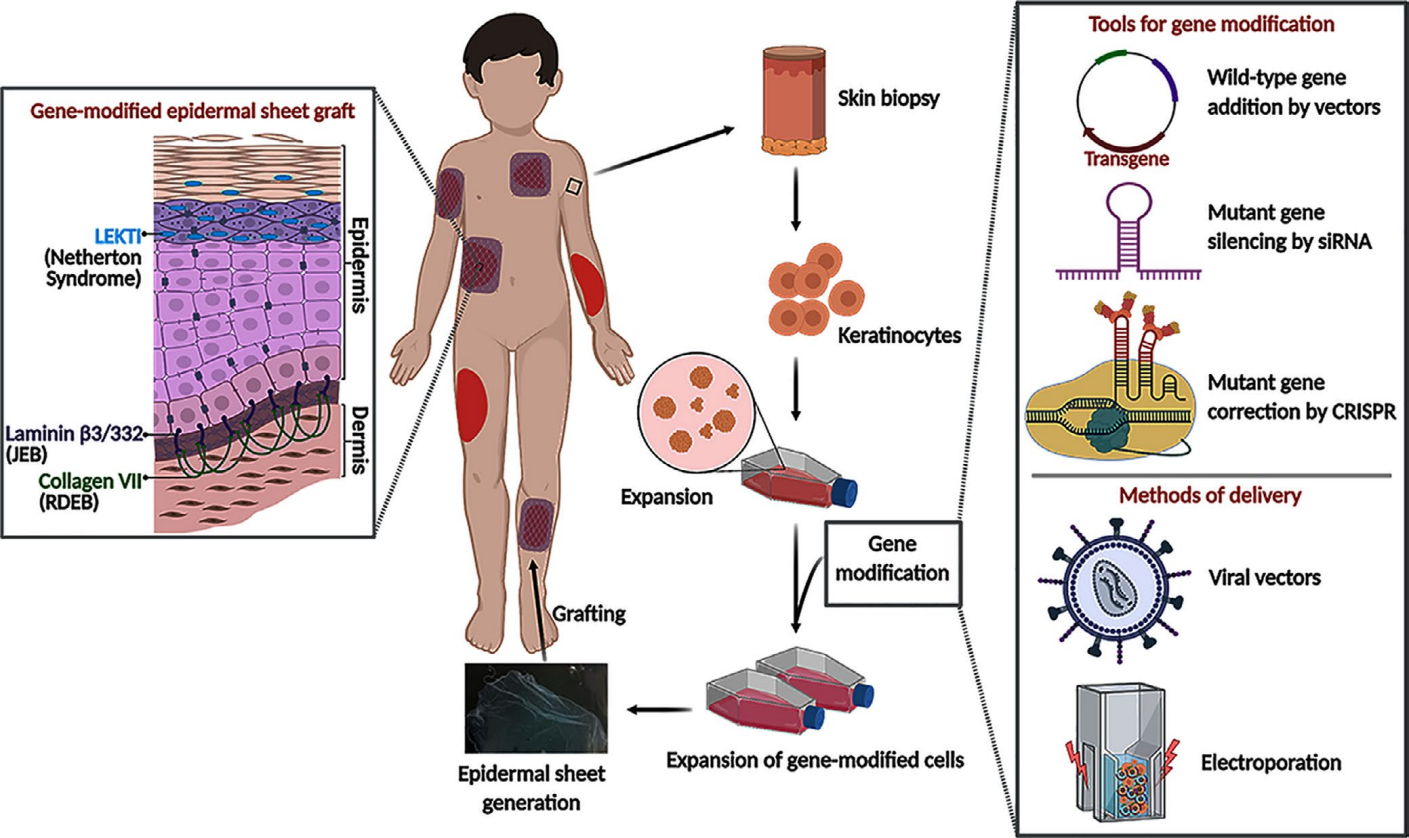
A flow chart of *ex‐vivo* epidermal sheet graft gene therapy. Patient cells isolated from a small skin biopsy are expanded, genetically modified, and cultured as epidermal sheet and then grafted back to the patients. 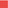
= indicates lesions;
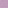
= indicates epidermal sheet graft on the lesions; LEKTI = lympho‐epithelial kazal‐type related inhibitor; JEB = junctional epidermolysis bullosa; RDEB = recessive dystrophic epidermolysis bullosa

*Ex vivo* gene therapy using genetically modified epidermal sheet grafts has been applied for the treatment of JEB and RDEB. This is because generalized blistering, recurrent wounds and fragile skin are so severe in these patients, and *ex vivo* autologous gene‐modified epidermal sheet therapy not only provides a long‐term functional gene/protein expression, but also covers wounds to support wound healing.^57^ In *ex vivo* epidermal sheet graft therapy, patients’ keratinocytes are isolated from a small skin biopsy, expanded, genetically modified, and cultured as epidermal sheet *in vitro* and then grafted back to the patients (Figure 1). Mavilio *et al*. carried out the first *ex vivo* gene‐modified autologous epidermal sheet therapy for JEB.^16^ In this study, keratinocytes including KSCs isolated from an adult JEB patient with laminin β3 (*LAMβ3*) deficiency were transduced with the retroviral vector carrying a wild‐type *LAM*β*3* cDNA. Transduced cells were then cultured as a epidermal sheet and grafted on the patient. A firmly adherent epidermis and restored laminin 332 protein expression were detected in the graft. Strikingly, transgene expression and restoration of normal epidermal‐dermal junction in the grafted area lasted for more than 6 years.^58^ This gene therapy strategy has recently been used on a 7‐year‐old child with JEB. The patient received ~0.85 m^2^ gene‐modified epidermal sheets containing nearly 1.6 × 10^7^ KSCs to cover the epidermal loss.^14^ Epidermal regeneration on the grafted regions was observed 1 month post‐grafting with normal skin morphology and without any blisters or wound. The regenerated epidermis also showed physiological levels of laminin 332, basal membrane with normal thickness, regular hemi‐desmosomes formation and wound healing for more than 4 years.^14^

Apart from these trials, there was an *ex vivo* epidermal sheet gene therapy for RDEB.^18^ In this trial, transgene *COL7A1* expression, functional C7 protein deposition and anchoring fibril formation in grafted areas were detected three months post‐grafting, but the expression of C7 protein declined/disappeared in 58% of grafted areas at 12 months post‐transplantation. *Ex vivo* epidermal sheet gene therapy was also applied for Netherton syndrome (NS).^17^ NS is an autosomal recessive disease caused by mutations in the gene *SPINK5* that encodes the protein LEKTI. The preclinical study on NS showed corrected skin morphology in the entire graft even though the grafts carried only a limited number of gene‐modified keratinocytes. As LEKTI is a secreted protein, it was speculated that the gene‐modified epidermal sheet graft may provide benefit not only at the site of grafting but also a generalized benefit around the grafting. Based on this, Di *et al*. carried out an *ex vivo* epidermal sheet graft gene therapy for NS. The expression of transgene *SPINK5* encoding protein LEKTI could be detected up to three months post‐grafting, but declined afterwards.^17^ Both preclinical experimental studies for RDEB and NS demonstrated stable successful transgene expression and functional correction in *in vivo* human:murine chimeric skin graft models. Why did transgene expressions decline in the trials for RDEB and NS used the protocols developed in preclinical studies?^59^, ^60^ Although the loss of long‐term transgene expression in these clinical studies might be due to overgrowth of non‐transgenic cells over transgenic cells, the loss of KSCs or KSC’s stemness during sheet culture could not be ruled out. As neither of the studies checked for KSC population after gene modification and prior to sheet grafting, the proportion of KSCs in the epidermal sheets in both trials was not clear.

The retention of genetically modified KSCs in cultured epidermal sheets determines the success of *ex vivo* genetically modified epidermal sheet therapy. *In vitro* cultivation of primary keratinocytes is of vital importance to maintain KSCs. In the skin, the basal layer of the epidermis contains KSCs and transient amplifying cells to meet the continuous demand of epidermal cell proliferation and differentiation.^61^, ^62^ KSCs have high self‐renewal capacity and give rise to transient amplifying cells, which have a limited proliferation ability of two to four generations before undergoing terminal differentiation.^13^, ^63^ Keratinocytes freshly isolated from the skin biopsies contain heterogeneous cell populations and can form three types of clones in culture including holoclones, meroclones and paraclones based on their morphology and size, and cells from holoclones have the highest proliferative capacity.^13^, ^61^ A holoclone is defined as a colony giving rise to less than 5% aborted colonies upon sub‐cultivation. Meroclones and paraclones do not have this feature.^64^, ^65^ In another words, the holoclone‐forming cells have hallmarks of a stem cell, while meroclone‐ and paraclone‐forming cells are transient progenitor cells.^14^ KSCs are currently unable to be separated from heterogeneous primary keratinocytes as a unique KSC marker could not be found. For this reason, primary keratinocytes with heterogeneous populations have to be used for generation of epidermal sheets. However, KSCs can be characterized by a panel of positive expression markers including integrins α1, α6, β1, and β6, delta N‐P63, and keratin 14 and negative expression markers that are only expressed in differentiated keratinocytes such as involucrin, loricrin, keratin 1 and transglutaminase 1. These markers can potentially be used to confirm KSC populations.^66^, ^67^, ^68^, ^69^

The properties of KSCs in primary cells can be affected by *in vitro* culturing environment. Two major culture systems have been used for culturing primary keratinocytes. One is the traditional culture system developed by Rheinwald and Green.^30^ This system employs Gibco Dulbecco's Modified Eagle Medium and Ham's F12 medium containing foetal calf serum and the supplements of hydrocortisone, tri‐iodothyronine, cholera toxin, epidermal growth factor, insulin and adenine.^30^ This culture system also requires co‐culturing keratinocytes with lethally irradiated or mitomycin C‐inactivated mouse embryonic fibroblasts (3 T3‐J2), known as feeder cells, as these feeder cells deposit basal lamina glycoproteins on the surface of culture dishes and secrete soluble factors into the culture media to support the attachment and colony formation of primary keratinocytes, preventing early differentiation of keratinocytes and overgrowth of human fibroblasts.^70^ It has been noticed that a fresh skin biopsy contains about 1–10% of KSCs, but only 0.1–1% KSCs may survive and behave as KSCs in the culture.^71^ This is because freshly isolated KSCs can undergo dissociation‐induced cell death called anoikis. A favourable surface for cell attachment and suitable microenvironment for cell growth and colony formation can alleviate and reduce anoikis, such as co‐culturing with feeder cells.^72^ Studies have showed that keratinocytes from holoclone can be propagated 20 – 180 times if they were cultured in a condition containing freshly irradiated 3 T3 cells with appropriate seeding density.^30^, ^64^ In the phase I NS trial, frozen‐irradiated 3 T3 cells instead of freshly prepared cells were used. As freeze‐thawing of irradiated 3 T3 cells caused tremendous stress to feeder cells in addition to irradiation, it resulted in batch‐to‐batch variation of cell viability and unpredictable and uneven seeding densities of 3 T3 feeder cells in cell cultures. The consequences of inappropriate use of feeder cells resulted in a low plating efficiency, poor holoclone formation, that is loss of KSC population, early differentiation of KSCs and overgrowth of fibroblasts in the culture.^17^ Although transient amplifying and differentiated keratinocytes can still form epidermal sheets, transgene expression would disappear when these cells undergo terminal differentiation and eventually die.

Serum‐free medium (SFM) without co‐culturing with feeder cells is another culture approach. This culture system uses the basal medium MCDB153 with low calcium concentration supplemented with bovine pituitary extract, epidermal growth factor, transferrin, insulin, hydrocortisone, monoethanolamine and phosphoethanolamine.^73^, ^74^ Feeder cells were replaced by coating culture surfaces with extracellular matrix such as collagen, fibronectin or laminin for keratinocyte attachment.^75^, ^76^, ^77^ SFM and feeder‐free culture avoids the use of animal materials such as foetal bovine serum and murine 3 T3 feeder cells, thereby reducing the risk of transmissible spongiform encephalopathies, and being suitable for translational clinical applications.^78^ Another advantage of using SFM is that unknown factors from serum and 3 T3 feeder cells can be controlled in studies. In addition, the lower calcium concentration (~0.06 mM) in SFM has been suggested to be beneficial for keratinocyte proliferation and prevention of cell differentiation.^79^, ^80^ However, there are conflicting opinions on feeder‐free culture for retention of KSCs. Studies showed that KSCs growing in feeder‐free culture might lose their stem cell properties such as limited proliferation ability and increased tendency towards differentiation and senescence.^81^, ^82^ In the trial of epidermal sheet graft therapy for RDEB, SFM and feeder‐free system was used and the outcomes showed a relatively short‐term transgene expression.^18^ It might be because the SFM and feeder‐free culture system was selective or favourite for transient amplifying cells but not KSCs, but this speculation remains unconfirmed as the trial for RDEB did not assess the KSC population after transduction and sheet culture. However, there are learning points from these studies, such as it is important to check the proportion of KSCs before grafting cells or epidermal sheet to the patients and this should be one of release criteria for investigational medicinal product (IMP).

### 1.6 Genetically modified fibroblasts

Intradermal injection of genetically modified dermal fibroblasts is another approach of *ex vivo* gene therapy for genodermatoses such as RDEB. Dermal fibroblasts are easy to isolate from the skin, require less complicated culture process compared to keratinocytes and can be extensively expanded *in vitro*.^83^, ^84^ As fibroblasts secrete C7 protein, the major component of dermal anchoring fibrils, intradermal injection of fibroblasts is, therefore, a relatively straightforward gene therapy approach for RDEB patients with the absence of C7 expression.^5^ A phase I/II clinical trial for RDEB utilized intradermal injection of gene‐modified autologous fibroblasts showed functional C7 expression and new anchoring fibrils formation.^85^ Another intradermal injection of autologous gene‐modified fibroblast for RDEB also revealed a significant increase in the expression of C7 at the site of injection for at least 12 months, although functional anchoring fibrils formation was not observed.^32^ However, it has to be aware that this therapy is unable to provide a long‐term therapeutic solution, and multiple intradermal injections of genetically modified fibroblasts would be required in order to maintain a durable therapeutic effect.

### 1.7 Future perspectives of gene therapy for genodermatoses

Since CRISPR/CAS9 was elucidated to be potential for genome editing in 2012,^86^ a large number of changes have been made on the CRISPR/Cas9 technology to improve its accuracy and efficiency, and CRISPR/Cas9 technology has currently become a powerful tool for editing genomes. Conventional CRISPR/Cas9 editing has proven to be indispensable for genome editing, but the major drawback of off‐target effect due to introduction of a double‐stranded DNA break limits its clinical application. Programmed base editing and prime editing have obviously advances compared to conventional CRISPR/Cas9. Both editors do not require the generation of double‐strand breaks as they use a catalytically inactive Cas9 variant instead. This change significantly reduces the frequency of off‐target activities in both editing systems. A number of studies have shown advances of base editing in precise genome editing and low off‐target effect compared to conventional CRISPR/Cas9. Nevertheless, base editing technologies do not yet represent a perfect editing tool for genome editing as it only corrects missense mutations, and is unable to target all genes due to protospacer adjacent motif (PAM) sequence preferences. Moreover, it has been reported that base editing induced genome‐wide off‐target deamination on both DNA and RNA, as well as unexpected nucleotide conversions.^87^, ^88^ Prime editing overcomes these limitations seen in conventional CRISPR/Cas9 and base editing by heavily modifying the Cas9 protein and the guide RNA, offering more targeting flexibility and greater editing precision. It has been estimated that prime editing might be able to correct nearly 89% of genetic variants known to be associated with human diseases. It looks prime editing holds great promise for clinical applications, but as the technology has been developed very recently (in 2019), there is much to do to prove that it is as general and robust as other genome editors. In addition, we have to be aware that there are potential challenges and risks involved in genome editing such as off‐target effect. It is important to make efforts to minimize or mitigate those issues of genome editors before subjecting to clinical application.^89^ For example, Base editors can be delivered as a form of ribonucleo‐protein (RNP) or mRNA which can reduce off‐target editing because both RNP and mRNA are rapidly degraded in cell cytoplasm.

In spite of recent advances in genome‐editing platforms, proof‐of‐concept experimental studies in human cells or tissues, for example, *in vitro* patient's cell models and *in vivo* murine:human chimeric skin graft models are essential. The gene correction efficiency, genocytotoxicity, off‐target effect and the efficacy of therapy should be evaluated in these models before translationally development of gene therapy strategies. Apart from gene correction, procedures involved in *ex vivo* gene‐modified therapy also need to be fully assessed including toxicity of electroporation for transferring genome editors into cells, culturing system for effective expansion and retention of KSCs and their stemness, and the approach and criteria for evaluation of KSC population.

In summary, there is no doubt that mutation correction using recently developed genome‐editing technology is the future direction of gene therapy for genodermatoses. Currently, *ex vivo* gene‐modified therapy for genodermatoses is still at its earlier stage, but it has the potential to treat those devastating genetic skin conditions in the near future.

## CONFLICT OF INTEREST

None declared.

## AUTHOR CONTRIBUTIONS

VJ contributes to writing and revision; EK contributes to writing and revision; WQ contributes review and comments; WLD contributes construction, writing and revision. All authors have read and approved the final manuscript.
